# Opioid-Induced Hypoglycemia in Diabetes: Dose-Dependent Effects of Methadone in a Complex Chronic Pain Patient

**DOI:** 10.7759/cureus.91389

**Published:** 2025-09-01

**Authors:** Omar Shazley, Christina Orr

**Affiliations:** 1 Department of Basic Sciences, Saint James School of Medicine, Kingstown, VCT; 2 Department of Diabetes, Metabolism, and Endocrinology, Vancouver Clinic, Vancouver, USA

**Keywords:** chronic pain management, hypothyroid, opioid use disorders, recurrent hypoglycemia, type 2 diabetes mellitus (dm)

## Abstract

Methadone is a widely used opioid for the management of chronic pain and opioid use disorder. Despite its safety profile, it is complicated by rare but significant side effects that can be easily overlooked in patients with multiple comorbidities. This case report details a challenging scenario of persistent hypoglycemia in a patient with a complex history of chronic pain syndrome, failed back surgery, and type 2 diabetes mellitus, who was maintained on high-dose methadone for pain control. Despite persistent hyperglycemia (glucose = 165-245 mg/dL, hemoglobin A1c = 7.0-8.1%) and the need for frequent insulin dose adjustments due to poor glycemic control, the patient experienced recurrent episodes of overnight hypoglycemia despite insulin administration for carbohydrate intake. The paradox of hyperglycemia with intermittent hypoglycemia was attributed to methadone use, with hypoglycemia episodes noted to be dose-dependent at methadone doses exceeding 40 mg daily. The patient’s endocrine comorbidities, including hypothyroidism and hypogonadism, further complicated glucose homeostasis and counter-regulatory responses. This case signifies the importance of maintaining a high index of suspicion of opioid-induced hypoglycemia in patients with diabetes and chronic pain, especially in the context of escalating methadone therapy. It highlights the need for individualized management strategies when multiple risk factors are present.

## Introduction

Diabetes mellitus (DM) is a complex metabolic disorder characterized by chronic hyperglycemia due to defects in insulin secretion and action [1]. It remains a leading cause of morbidity and mortality worldwide, associated with significant complications, comorbidities, and economic burden [1]. Its prevalence steadily increases, necessitating effective management strategies combining lifestyle modification, pharmacological intervention, and individualized care to minimize complications [1].

Renal impairment is a significant complication of DM [1]. It contributes to morbidity and mortality among affected individuals [1]. Chronic kidney disease (CKD) is one of the most common comorbidities in adults with diabetes, with diabetes accounting for a significant proportion of new cases of renal failure and end-stage kidney disease requiring dialysis or transplantation [1]. Impaired renal function further complicates glycemic management and increases the risk of hypoglycemic episodes, necessitating close monitoring and individualized therapy in this population [1].

Methadone is a long-acting μ-opioid receptor agonist commonly prescribed for chronic pain management and opioid use disorder. While it is effective in these roles, its use is associated with various metabolic side effects, including alterations in glucose homeostasis [2]. Notably, methadone has been implicated in rare but clinically significant cases of hypoglycemia, particularly at higher doses [2]. The mechanism underlying opioid-induced hypoglycemia is not fully elucidated but may involve suppression of counter-regulatory hormone responses and altered hepatic glucose output [2].

Patients with DM are uniquely vulnerable to disturbances in glucose regulation, and the co-administration of high-dose opioids, such as methadone, can further complicate glycemic control. Persistent hyperglycemia is a hallmark of poorly controlled diabetes, yet the paradoxical occurrence of recurrent hypoglycemic episodes, particularly overnight, presents a significant management challenge [3]. This apparent contradiction is clinically significant, as it necessitates frequent insulin dose adjustments and close monitoring to avoid both hyperglycemia and hypoglycemia extremes.

Endocrine comorbidities, including hypothyroidism and hypogonadism, further complicate the clinical picture by impairing counter-regulatory responses to hypoglycemia and altering insulin insensitivity [4,5]. Thyroid hormone deficiency is associated with reduced hepatic glucose production and impaired gluconeogenesis, while hypogonadism may contribute to insulin resistance and altered body composition [4,5]. Together, these factors create a complex interplay that disrupts glucose homeostasis and increases the risk of both hyperglycemia and hypoglycemia in patients with diabetes and chronic pain syndromes.

Given the increasing prevalence of chronic pain and opioid use in patients with diabetes, it is essential to recognize the potential for opioid-induced hypoglycemia and the added challenges posed by endocrine comorbidities [6]. We present a case of a 61-year-old man with chronic pain syndrome, type 2 DM, hypothyroidism, and hypogonadism presenting with persistent hyperglycemia and dose-dependent hypoglycemic episodes in the setting of high-dose methadone therapy.

## Case presentation

A 61-year-old male with a complex medical history, including chronic low back pain, failed back surgery syndrome, chronic intractable pain, type 2 DM, hypothyroidism, and hypogonadism, was referred for ongoing management of both pain and glycemic control. His history of chronic pain was complicated by lumbar radiculopathy, lumbar postlaminectomy syndrome, vertebral compression fractures, and multiple musculoskeletal injuries (left ankle). Over the years, he underwent various pain management strategies, including physical therapy, spinal cord stimulator trial, and surgeries (lumbar spine, right shoulder, left ankle fixation, and umbilical hernia repair).

For chronic pain, the patient was initially managed with conservative measures throughout the years, including physical therapy, chiropractic, and acupuncture. He has been on chronic opiate therapy for years, starting with escalating doses of oxycodone 15 mg daily and OxyContin 20 mg twice daily in April 2014, and has tried several other adjuvant medications as well. He later transitioned to Suboxone and high-dose methadone. By early 2025, his methadone dose had reached 95 mg daily. In August 2018, he transitioned to Suboxone 16 mg daily. After efforts to taper off Suboxone in January 2019, he experienced drug-induced anxiety with suicidal ideation. He was subsequently prescribed Cymbalta 60 mg daily; however, this medication worsened his side effects and failed to adequately provide pain relief, resulting in him transitioning back to low-dose oxycodone. The patient expressed reluctance to restart on oxycodone and was started on Namenda 5 mg twice daily in March 2019, but noted it did not provide the immediate gratification the short-acting opiate provided. He would transition back to oxycodone 10 mg four times daily by May 2019, with additional OxyContin 20 mg two times daily in December 2019. Despite replacing OxyContin with fentanyl 75 mcg daily patches on March 2021, he continued to report severe, persistent pain particularly in his lower back and upper neck (from landing on his back due to a fall and whiplash in a motor vehicle accident), right shoulder (November 2016), and left ankle (type III open fracture lateral malleolus of the left fibula due to fall from ladder May 2021), with pain scores often 7/10 without medication. His medical history was significant for lumbosacral neuritis and spondylosis without myelopathy, with his pain noted in the lower lumbar region with radiating dysesthesias down the left mid and right leg, with occasional neuropathy and tingling down his legs. He has tried numerous spinal injections, including radiofrequency ablations, with variable degrees of success over the years. His pain management was further complicated by episodes of medication nonadherence, opioid contract breaches due to a history of alcohol use, and a history of intentional opioid overdose.

**Figure 1:**
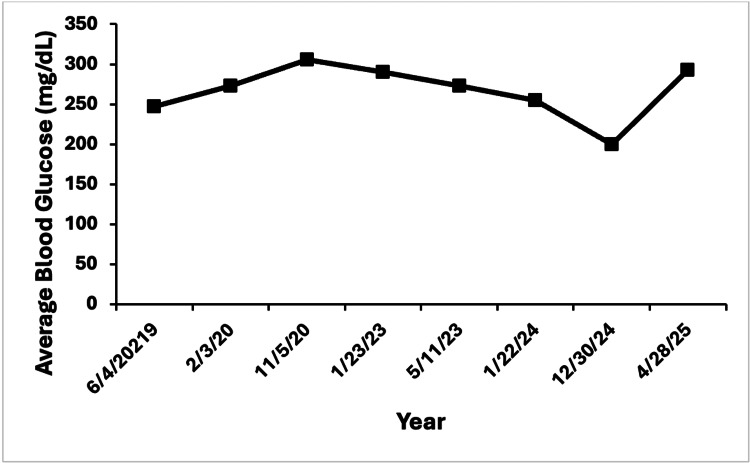
Longitudinal trend of average blood glucose levels between 2019 and 2025. Illustration of persistent hyperglycemia despite changes in insulin therapy.

The patient’s diabetes management was equally challenging. His glycemic control was closely tracked using a glucometer and, for limited periods, with a Dexcom (San Diego, CA) continuous glucose monitor (CGM). Sensor data from his CGM were available only for brief periods (May and September 2023), with the remainder of glycemic data obtained from self-monitored glucometer readings recorded during his visits to endocrinology. The data shown in Table 1 reveal a pattern of persistent hyperglycemia with marked glycemic variability and intermittent hypoglycemic episodes, particularly overnight. Notably, time-in-range percentages (70-180 mg/dL) were consistently low, ranging from 14% to 33% on sensor data, and coefficients of variation (CV%) were elevated (43-44%), reflecting glucose fluctuations. Glycemic variability remained high with CV% > 40% during sensor-monitored periods, and average daily blood glucose (BG) levels consistently above 245 mg/dL across all years (Figure 1). Analysis of average BG by time of day further demonstrated significant elevations in postprandial and nocturnal periods (Table 2). Despite persistent hyperglycemia with average BG levels ranging from 165 to 300 mg/dL (reference range: 70-99 mg/dL) and elevated hemoglobin A1c values between 7.0% and 9.6% (reference range: 4.0-5.6%), he experienced recurrent episodes of overnight hypoglycemia (Table 2). These episodes were consecutively documented by endocrinology, with the patient and provider both noting that hypoglycemia occurred despite appropriate insulin dosing for carbohydrate intake. In response, his insulin regimen was frequently adjusted with reductions in both basal (Lantus or Levemir) and bolus (Humalog or Novolog) doses as hypoglycemic episodes became more frequent.

**Table 1:** Temporal correlation of methadone dosage, insulin use, and glycemic control. ª Not applicable.

Date	Methadone dose (mg)	Total daily insulin (units)	Average blood glucose (mg/dL)	Time in range (%)	Coefficient of variation (% CV)	Hemoglobin A1c (%)	Hypoglycemic episode frequency
6/4/2019	0	~120	247	-ª	-	-	Occasional
2/3/2020	0	~120	273	-	-	-	Rare
11/5/2020	0	~120	306	-	-	-	Occasional
1/23/2023	0	~120	290	-	-	-	Occasional
5/11/2023	0	~90	273	24	44	-	Occasional
9/18/2023	0	~90	245	33	-	7.9	Occasional
1/22/2024	0	~80	255	16	-	8.4	Occasional
12/30/2024	95	~60	200	-	-	8.5	Frequent
4/28/2024	45	~56	293	14	43	8.5	Frequent

**Table 2:** Average self-monitored blood glucose levels by time of day. ª Not applicable.

Date	Morning (mg/dL)	Afternoon (mg/dL)	Evening (mg/dL)	Night (mg/dL)
5/23/2019	71	384	152	321
6/4/2019	72	273	111	205
2/3/2020	62	210	257	200
11/5/2020	175	329	261	346
5/12/2021	250	278	336	207
1/23/2023	275	249	293	-
5/10/2024	69	-ª	-	235
12/21/2024	118-179	157	111	-

His endocrinologist noted a clear temporal relationship between the escalation of methadone therapy and the onset of dose-dependent hypoglycemia, particularly at doses exceeding 40 mg daily. Since starting methadone, the patient has not used a CGM, limiting the granularity of nocturnal hypoglycemia detection during the period of highest opioid risk. He relied on self-monitoring his glucose averages with a glucometer. He regularly recorded and reviewed his blood glucose averages at his annual appointments with endocrinology. The patient periodically declined both an insulin pump and sensor, believing his diabetes was stable and noting that his wife, who uses a CGM, often found it alarming for low values. Despite encouragement from both his wife and endocrinologist, he remained uninterested in trying a sensor. He eventually expressed interest in starting on a CGM during his most recent visit with his endocrinologist, as his glycemic levels became more erratic with increased frequency of both hypo- and hyperglycemia. A detailed timeline displaying the relationship between methadone, insulin requirements, and hypoglycemic episodes is presented in Table 3. The patient required frequent insulin dose adjustments due to this paradoxical combination of persistent hyperglycemia and intermittent hypoglycemia.

**Table 3:** Timeline for methadone dosage, insulin requirements, and hypoglycemic episodes.

Date	Methadone dose (mg)	Total daily insulin (units)	Average blood glucose (mg/dL)	Hypoglycemic episodes per month	Hemoglobin A1c (%)
2022	Oxycodone/fentanyl	120-140	245-330	Rare	8.5-9.6
2023	Oxycodone/fentanyl	100-120	255-290	Occasional	7.8-8.2
2024	Transition period	80-100	200-255	Increasing	7.5-8.1
2025	Methadone 95 mg -> 60 mg	50-70	165-245	3-4/month	7.0-7.8

Laboratory evaluations were performed throughout the patient's management, with data collected from 1/3/2013 to 4/28/2025. The results summarized in Table 4 represent cumulative averages across this time. Total testosterone levels ranged from 234 to 306 ng/dL (reference range: 250-1,100 ng/dL), with free and bioavailable testosterone at or below the lower end of the normal range. TSH values were intermittently elevated, reaching as high as 8.3 uIU/mL (reference range: 0.270-4.20 uIU/mL), consistent with undertreated hypothyroidism. Other laboratory findings included fluctuating hematocrit and albumin levels and sex hormone-binding globulin within normal limits. These comorbidities further complicated the patient’s glucose homeostasis and contributed to the difficulty in achieving stable glycemic control.

**Table 4:** Comprehensive metabolic laboratory parameters during persistent hyperglycemia and endocrine dysfunction. Cumulative values represent averages from 1/3/2013 to 4/28/2025.

Parameter	Patient's range	Reference range
Hemoglobin A1c (%)	7.0-9.6	4.0-5.6
Hematocrit (%)	36-41	38.5-50.0
Glucose (mg/dL)	165-245	70.0-99.0
Sex hormone-binding globulin (nmol/L)	24-38	22.0-77.0
Total testosterone (ng/dL)	234-306	250.0-1,100.0
Free testosterone (pg/mL)	41-102	46.0-224.0
Bioavailable testosterone (ng/dL)	98-210	110.0-575.0
Albumin (g/dL)	3.9-4.7	3.60-5.10
Thyroid-stimulating hormone (uIU/mL)	2.1-8.3	0.270-4.200

**Figure 2:**
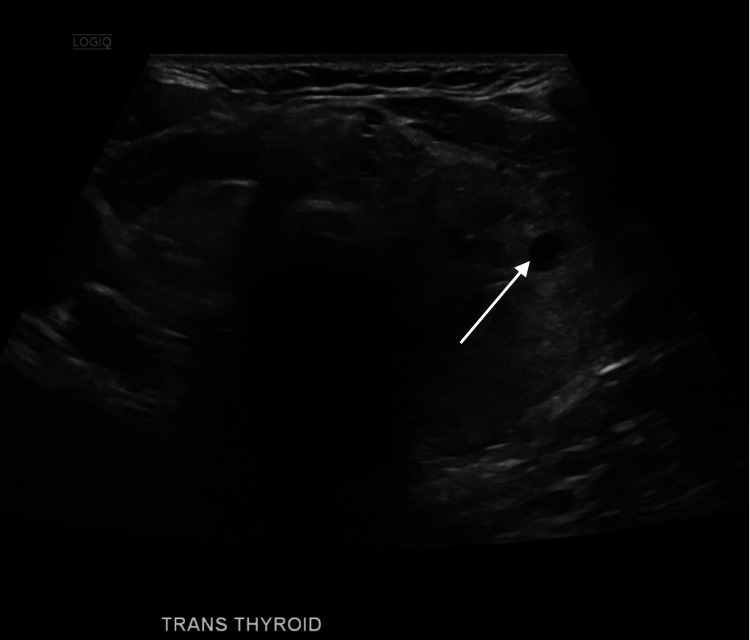
Transverse thyroid ultrasound illustrating nodule enlargement (March 2020). Ultrasound image demonstrating progressive enlargement of a thyroid nodule (arrow) within a multinodular goiter. The transverse view highlights a dominant nodule with well-defined margins and internal heterogeneity, consistent with the patient’s history of thyroid dysfunction.

Imaging studies over the years documented the sequelae of his chronic pain and comorbidities. Lumbar and thoracic spine MRI showed multilevel degenerative disc disease, vertebral compression fractures, and postoperative changes. His recent thyroid ultrasound in March 2020 revealed a multinodular goiter with progressive enlargement of nodules (Figure 2), while musculoskeletal imaging confirmed recurrent fractures and multiple surgical interventions.

Throughout his clinical course, the patient experienced multiple hospitalizations for acute pain exacerbations, opioid overdose, including a suicide attempt with 100 tablets of oxycodone 15 mg, and episodes of acute toxic encephalopathy. Pain management efforts included trials of Suboxone and fentanyl patches, with each transition closely followed by changes in glycemic control and insulin requirements. Social and psychological stressors, including substance use, medication theft, and repeated injuries, further complicated his care.

## Discussion

This case illustrates the complex management of persistent hypoglycemia in a patient with type 2 DM and chronic pain, whose glycemic control was further complicated by high-dose methadone therapy and multiple endocrine comorbidities. The paradoxical occurrence of both persistent hyperglycemia and recurrent overnight hypoglycemia signifies the clinical challenges posed by opioid-induced metabolic disturbances. While this combination is unusual, a similar pattern of persistent high and low blood sugars can sometimes be seen in patients treated with insulin or sulfonylureas, particularly in the setting of mismatched dosing or renal impairment. However, our patient's renal function remained normal throughout management, suggesting other factors contributed to his blood glucose fluctuations. It highlights the need for heightened awareness and individualized management in this vulnerable population.

The mechanisms underlying methadone-induced hypoglycemia remain incompletely understood but are likely multifactorial. Methadone, a synthetic μ-opioid receptor agonist, is widely used for chronic pain and opioid use disorder. While most opioids are associated with minimal effects on glucose homeostasis, methadone and tramadol have been linked to a significantly increased risk of hypoglycemia at higher doses [2,7,8]. Proposed mechanisms include direct effects on the pancreas, possibly mediated through μ-opioid receptors, suppression of counter-regulatory hormones such as glucagon and epinephrine, and central nervous system effects via serotonergic pathways [2,7]. Animal studies demonstrate that methadone can lower blood glucose dose-dependently, likely through increased glucose utilization in peripheral tissues and inhibition of hepatic gluconeogenesis [2,9]. These effects appear to be specific to methadone and tramadol, as other opioids such as fentanyl, oxycodone, and morphine do not exhibit the same hypoglycemic risk [2,7,8].

A robust dose-response relationship between methadone and hypoglycemia has been demonstrated in both clinical and experimental settings. Retrospective analyses have shown that methadone exposure is associated with a significant reduction in average minimum daily blood glucose and a marked increase in the incidence of hypoglycemia, particularly at doses exceeding 40 mg daily [7,10,11]. In our patient, the temporal correlation between methadone dose escalation and the onset of frequent hypoglycemic episodes was apparent, as evidenced by the reduction in insulin requirements and the increase in hypoglycemia frequency after methadone initiation (Table 4). This pattern is consistent with prior case series and observational studies, which have reported that rapid methadone dose escalation can precipitate hypoglycemia, often necessitating dose reduction or opioid rotation [11,12].

The interaction between methadone and glucose metabolism creates a unique challenge in patients with diabetes. Despite persistent hyperglycemia, reflected in average blood glucose levels consistently above 245 mg/dL and elevated hemoglobin A1c values, the patient experienced recurrent episodes of overnight hypoglycemia, even when insulin was appropriately dosed for carbohydrate intake. This paradox is supported by emerging literature, which suggests that opioids may lower blood glucose in patients with baseline hyperglycemia, possibly by enhancing glucose uptake in peripheral tissues and impairing counter-regulatory responses [13,14]. Reviewing the patient's dietary habits provides critical context for these glycemic patterns. He regularly skips breakfast and consumes most of his calories and high-carbohydrate snacks (such as ice cream, cheese, crackers, and pretzels) after dinner. He often takes additional insulin at bedtime for elevated glucose, resulting in lower fasting levels. Despite efforts to curb nocturnal snacking due to weight gain and inactivity, this likely contributed to the nocturnal hyperglycemia and morning hypoglycemia, as observed in Table 2. This highlights the impact of lifestyle factors on glycemic variability. The need for frequent insulin dose adjustments and the reduction in total daily insulin requirements signify the clinical impact of methadone-induced hypoglycemia and the importance of vigilant glycemic monitoring in this population [7,12].

The patient’s endocrine comorbidities, including hypothyroidism and hypogonadism, further complicated glucose homeostasis. Hypothyroidism is known to impair hepatic gluconeogenesis and glycogenolysis, delaying recovery from hypoglycemia and increasing susceptibility to glucose fluctuations [15,16]. Hypogonadism, characterized by low testosterone levels, is associated with insulin resistance and altered glucose metabolism, which may exacerbate glycemic instability [17]. These hormonal disturbances likely contributed to the patient’s difficulty in achieving stable glycemic control and increased his risk for both hyperglycemia and hypoglycemia. The presence of multiple comorbidities is itself a strong independent risk factor for hypoglycemia-related complications, as demonstrated by recent large-scale studies [18].

The limitations of CGM during the period of highest opioid risk, due to the discontinuation of sensor use after methadone initiation, highlight the ongoing challenges in detecting nocturnal hypoglycemia in this population. CGM, while valuable for assessing glycemic variability and time-in-range, has inherent limitations, including sensor delays and accuracy issues, which may delay the recognition and management of hypoglycemic episodes [19]. The lack of granular nocturnal data during methadone therapy signifies the importance of frequent self-monitoring of blood glucose and the need for clinical vigilance in patients receiving high-dose opioids.

There are currently no established guidelines for the management of methadone-induced hypoglycemia; however, case reports and certified recommendations suggest several strategies to mitigate risk, including dose reduction, dose splitting (i.e., twice daily doses), and opioid rotation to agents with lower hypoglycemic potential, such as buprenorphine [12,20]. In our patient, splitting the methadone dose or transitioning to buprenorphine could be considered to reduce the risk of dose-dependent hypoglycemia. In addition, careful monitoring of glucose levels, particularly during periods of opioid dose adjustment, is essential to prevent severe hypoglycemic episodes [7,12]. Patient education regarding hypoglycemia awareness and the importance of regular glucose monitoring is also critical, especially in individuals with multiple comorbidities and complex medication regimens [18].

This case is limited by the absence of CGM data during the period of methadone dose escalation, which may have resulted in under-detection of nocturnal hypoglycemia. In addition, the year-to-year trend analysis of hemoglobin A1c is limited by gaps in interim testing, with some periods lacking regular measurements. This restricts the ability to observe and interpret longitudinal glycemic control changes. Routine and systematic hemoglobin monitoring is recommended for future studies to enhance the continuity and reliability of metabolic trend assessment. The lack of counter-regulatory hormone measurements during hypoglycemic episodes limits our ability to fully elucidate the underlying pathophysiology. Future studies should prospectively evaluate the effects of methadone on glucose metabolism in patients with diabetes and chronic pain, using CGM and comprehensive endocrine assessments, to better characterize the risk factors and mechanisms of opioid-induced hypoglycemia.

This case report was prepared in accordance with the CARE (CAse REport) guidelines to ensure transparency and completeness of clinical reporting. Adherence to frameworks such as STROBE (Strengthening the Reporting of Observational Studies in Epidemiology) or EQUATOR is recommended for further observational or larger studies to enhance clarity and reproducibility.

## Conclusions

This case highlights the importance of recognizing methadone-induced hypoglycemia as a significant, dose-dependent adverse effect in patients with diabetes and chronic pain, particularly those with multiple endocrine comorbidities. The paradoxical combination of persistent hyperglycemia and recurrent hypoglycemia, in the setting of high-dose methadone therapy, presents unique challenges for glycemic management and signifies the need for individualized, multidisciplinary care. Notably, methadone-induced hypoglycemia exemplifies a case of personalized medicine, highlighting the necessity for close and ongoing monitoring to tailor management strategies to each patient's unique risk profile and therapeutic response. Clinicians should maintain a high index of suspicion for opioid-induced hypoglycemia, monitor glucose levels closely during methadone dose adjustments, and consider alternative pain management strategies in high-risk patients. Further research is needed to establish evidence-based guidelines for the prevention and management of this underrecognized complication.
